# Development of Visibly Opaque Polyolefin Sheets While Preserving Infrared-Light Transparency

**DOI:** 10.3390/mi16020178

**Published:** 2025-01-31

**Authors:** Md. Saiful Hoque, Mehnab Ali, Xiaoruo Sun, Asad Asad, Patricia I. Dolez, James David Hogan, Dan Sameoto

**Affiliations:** 1 Department of Mechanical Engineering, University of Alberta, Edmonton, AB T6G 2R3, Canada; hoque1@ualberta.ca (M.S.H.);; 2 Department of Human Ecology, University of Alberta, Edmonton, AB T6G 2N1, Canada

**Keywords:** LLDPE sheets, pigments, infrared transparency, visibly opaque, micro-structure, IR tuning

## Abstract

This study focused on developing pigmented linear low-density polyethylene (LLDPE) sheets while preserving their mechanical properties and infrared (IR) transparency. Six pigments—ZnO, ZnS, TiO_2_, FeO yellow, FeO light brown, and FeO dark brown—were each mixed with polyethylene (PE) wax in a 1:1 ratio and blended with LLDPE at concentrations of 1, 3, and 5 wt%. Tensile strength tests showed minimal changes at lower pigment concentrations, with values near that of pure LLDPE (14 MPa), and slight reductions at 5 wt%. IR transparency tests, conducted using both direct and reflected heat sources, showed that white-pigmented sheets maintained over 85% transparency, while colored pigments exhibited slightly reduced IR transmittance, ranging from 70% to 91%. Fourier Transform Infrared Spectroscopy (FTIR) analysis confirmed that the critical IR transparency range of 8–12 μm remained unaffected with both pure and pigmented sheets. On the other hand, ultraviolet–visible (UV–VIS) testing showed that white-pigmented sheets experienced enhanced visible-light absorption with increasing pigment concentration and thickness, while color-pigmented sheets exhibited high opacity. Additionally, micro-structuring was performed on the LLDPE sheets to further modify their IR properties, which resulted in effective scattering of IR radiation. These findings highlight the potential of pigmented LLDPE sheets for applications requiring both visual opacity and IR transparency, such as thermal management and camouflage, as well as applications requiring tunable IR properties.

## 1. Introduction

A polyolefin is a polymer produced from an olefin or alkene as a monomer. In organic chemistry, an alkene or olefin is an unsaturated chemical molecule containing at least one carbon-to-carbon double bond [1]. Polyolefins include PE (low-density, high-density, and linear low-density), polypropylene (PP), and polybutene (PB) (polybutene-1 and polyisobutylene) [2]. PE and PP are the most commonly used commercially available polyolefins.

LLDPE is a low-density (0.915 to 0.930 g/cm^3^), semi-crystalline polymer with short branches on its main molecular chains [3]. This means that these linear molecules do not become entangled as easily as low-density polyethylene (LDPE) and high-density polyethylene (HDPE). LLDPE has many advantages and is well suited for thin-sheet applications. Its excellent attributes like puncture resistance, good flexibility, resistance to oxidation, high impact strength, stress-cracking resistance, and UV resistance make it desirable for various packaging, agricultural, medical, and industrial applications [4,5,6,7,8,9].

LLDPE is often compounded with different fillers and additives to enhance and tailor its capabilities for various applications. In the packaging industry, LLDPE is widely compounded with additives such as antioxidants, slip agents, and anti-block agents to improve its performance when making sheets, bags, and other packaging products [10,11,12]. For instance, Dadbin et al. explored replacing the PE multi-layer sheets used in the food packaging industry with single-layer LLDPE nanocomposite sheets [13]. They reported that the addition of organoclay, even at a low level (below 5 per hundred resin (phr)), to LLDPE had a significant effect on the barrier properties of the sheets. The researchers decreased the oxygen permeability by 50% by adding only 3 phr of nanoclay to the blend. Panrong et al. added Green tea (GT) and thermoplastic starch (TPS) from native starch (NS) and acetylated starch (AS) with different degrees of substitution to LLDPE to develop TPS-GT LLDPE sheets [14]. These sheets showed high efficacy as active eco-friendlier food packaging, with enhanced stability of meat and oil-based food products.

Similarly, LLDPE can be compounded with UV stabilizers and other additives to improve its resistance to environmental degradation [15], resulting in its application in the agriculture sector as greenhouse covers, silage bags, and mulch sheets [16,17,18]. Korol et al. investigated the recycling potential of waste from the recycling of LLDPE sheets used for agricultural silage [19]. They modified LLDPE with the addition of 2.5 wt% of commercially available compatibilizers. They found that adding compatibilizers such as maleic anhydride-grafted compounds, ethylene–vinyl acetate, and styrene–ethylene–butadiene–styrene improved the mechanical properties of LLDPE waste, increasing its strength while reducing its stiffness. Seven et al. described an insecticide-releasing plastic sheet produced by incorporating deltamethrin-loaded halloysite nanotubes into LLDPE sheets for agricultural covering purposes [16]. The nanocomposite sheet was observed to repel mature aphids and kill young aphids and thrips. In the construction industry, LLDPE is used as a geomembrane material for lining landfills, reservoirs, and other water containment systems [20,21,22]. It is compounded with additives such as carbon black and antioxidants to improve its resistance to UV radiation, weathering, and chemical degradation [23,24,25,26,27]. In the medical industry, LLDPE is compounded with biocompatible additives to generate products such as medical tubing, bags, and antibacterial containers [28]. For instance, Harun et al. investigated the efficiency of LLDPE nanocomposites loaded with titanium dioxide (TiO_2_) nanoparticles, zinc oxide (ZnO) nanoparticles, or a combination of both in a 1:3 ratio against two representatives of multidrug-resistant (MDR) pathogens, namely methicillin-resistant *Staphylococcus aureus* (MRSA) and *Klebsiella pneumonia* [29]. In the case of MRSA, they observed reductions in bacterial growth of 67.65% for LLDPE/TiO_2_, 100% for LLDPE/ZnO, and over 98% for LLDPE/TiO_2_/ZnO nanocomposites. The LLDPE/ZnO composite achieved 83% bactericidal efficiency against K. pneumoniae. They attributed this bactericidal function of the nanocomposites to the ZnO nanoparticle’s photocatalytic activity, zinc ion release, and reactive species generation. The composite made with the highest ZnO ratio tested (10 wt%) was found to be the most effective in killing both types of pathogens.

In recent years, polyolefins, particularly LLDPE, have gained significant attention for their tunable optical properties and potential applications in radiative cooling and thermal management. As passive cooling systems become more essential in energy-saving technologies, the ability to produce materials that maintain infrared (IR) transparency while offering visible opacity is crucial for applications such as building materials, clothing, and food packaging [30,31,32,33,34,35,36,37]. For instance, to produce LLDPE fibers with improved radiative cooling capabilities, Alberghini et al. came up with a multi-step production procedure to engineer the structures at the fiber, yarn, and fabric levels [31]. Their method involved a complex sequence of melt extrusion of LLDPE to produce fibers, which also included an additional step of spin-dyeing for coloration. The final product achieved high infrared transparency and visible opacity, resulting in radiative cooling of up to 5 °C below ambient temperature. Similarly, Peng et al. achieved radiative cooling using nanoporous PE microfibers [38]. They used paraffin oil as a solvent to dissolve PE before extruding it to form fibers with microscale diameters. After fiber production, methylene chloride was used to extract the oil phase from the PE fibers, which created nanopores in the fibers. The fabric produced with the nanoporous PE microfibers exhibited high IR transparency while maintaining visible opacity. This led to a skin temperature reduction of 2.3 °C compared to conventional cotton fabrics. The nanoporous PE fiber fabric also demonstrated durability after five domestic washing cycles and had a water vapor transmission rate of 0.023 g cm^−2^h^−1^, which was superior to traditional cotton. However, the manufacturing procedures for these materials are intricate, involve expensive equipment, and require careful control over several steps and rigorous material structure engineering. Scalability and economic viability may be limited by such complexity, emphasizing the need for more straightforward production processes to obtain the required optical features in infrared and visible light.

This study addressed several limitations in existing methodologies for producing visibly opaque, IR-transparent polyolefin sheets. While previous works have demonstrated the potential of such materials for radiative cooling and thermal management [30,31,32,33,34,35,36,37], they have often relied on complex, multi-step processes that limit scalability and economic viability. Our approach offers a simplified, one-step custom compounding method that not only achieves the desired optical properties but also maintains the mechanical performance of LLDPE. Furthermore, the ability to easily micro-structure these sheets for enhanced scattering is a unique feature that was not prominently addressed in previous studies. This work thus represents a significant step forward in making IR-transparent, visibly opaque polyolefin sheets more accessible for a wide range of applications, from building materials to textiles and packaging, where both aesthetic appearance and thermal management are crucial.

## 2. Materials and Methods

### 2.1. Materials

Linear low-density polyethylene (LLDPE, MKCH0863; melt index: 1.0 g/10 min (190 °C/2.16 kg)) was purchased from Sigma-Aldrich (Oakville, ON, Canada). Zinc oxide (ZnO, powder, <5 μm, UPS-2 grade), titanium dioxide (TiO_2,_ powder, 1–150 nm, FCC grade), and zinc sulfide (ZnS, powder, 10 µm) were acquired from Sigma-Aldrich (Oakville, ON, Canada). Iron oxide yellow (FeO, powder, yellow 930 dark, synthetic, >3 nm), iron oxide light brown (FeO, powder, brown 610 light, synthetic, >3 nm), and iron oxide dark brown (FeO, powder, brown 686 dark, synthetic, >3 nm) were purchased from Kremer Pigmente Inc. (Aichstetten, Germany). Polyethylene wax powder (low melting point of 105 °C) was purchased from Chemical Store Inc. (Clifton, NJ, USA).

### 2.2. Methods

#### 2.2.1. Sample Fabrication

**Figure 1:**
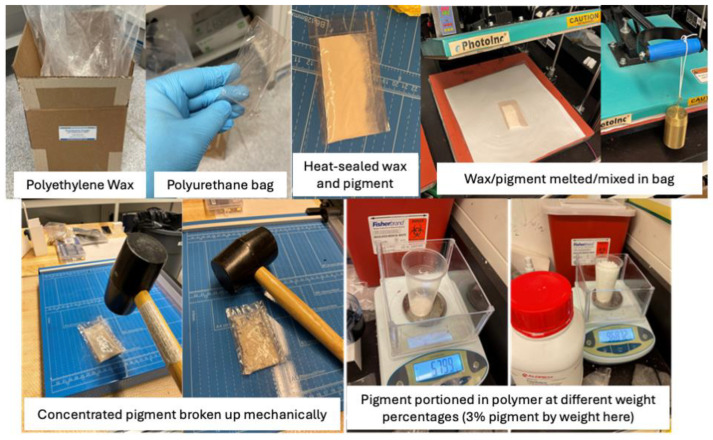
Step-by-step process of master-batch preparation.

Master batches with the pigments were prepared by mixing each pigment with the polyethylene wax powder in a 1:1 ratio by weight, forming 50 g batches. The pigment–wax mixtures were sealed in high-temperature-resistant polyurethane pouches and heat-pressed at 190 °C until the mixtures fully melted. After melting, the mixture pouches were flipped and heat-pressed again, repeating the process a total of three times to ensure uniform melting. The melted mixtures were then cooled to ambient temperature and crushed into master-batch granules. The process of master-batch preparation is illustrated in Figure 1.

For each blend, a specific amount of the pigment–wax master-batch granules was combined with LLDPE pellets to obtain the desired concentration. For example, a 3 wt% ZnS-LLDPE blend was made by mixing 6 g of the pigment–wax mixture (3 g of pigment and 3 g of wax) with 94 g of LLDPE. Similarly, a 5 wt% FeO yellow–LLDPE blend was created by mixing 10 g of the pigment–wax mixture with 90 g of LLDPE. The use of the polyethylene wax powder ensured a uniform dispersion of the pigments with the LLDPE. A total of 18 LLDPE–pigment blends were produced (Table 1), covering six pigments at three different concentrations.

The LLDPE–pigment blends were extruded into filaments using a Wellzoom B single-screw desktop extruder (Shenzhen, China). The extruded polymer strands were pulled using a gravity-fed pulling system, cooled at ambient temperature, and wound onto spools. The extrusion parameters utilized for filament fabrication are provided in Table S1 (in the Supporting Information). Figure S1 (in the Supporting Information) displays the filaments produced for the pigmented LLDPE concentrations.

The extruded filaments from each pigmented LLDPE blend were cut into small pieces and melted using a 10 ton hydraulic heat press (CH2042-1, China) at 190 °C under pressures of 2, 4, and 6 bars to form sheets with thicknesses of 150, 100, and 50 μm, respectively. The sheets were cut into 1 × 1-inch squares for each of the 18 blends. Additionally, pure LLDPE sheets of the same thicknesses were prepared for comparison. The fabrication process is illustrated in Figure S2 (in the Supporting Information), and pictures of the pigmented sheets are shown in Figure S3 (in the Supporting Information).

#### 2.2.2. Characterization

##### Tensile Strength

Mechanical tests were conducted on the extruded filaments. For each of the 18 pigmented LLDPE blends and pure LLDPE, 25 mm gauge-length filaments were cut and installed between the pneumatic clamps of an Instron 5960 machine (Illinois Tool Works Inc., Norwood, MA, USA) with a 50 N load cell. The filaments were stretched at 25 mm/min until failure, with four samples tested per blend. The tensile strength, Young’s modulus, and elongation at break were recorded for each sample.

##### Fourier Transform Infrared Spectroscopy Analysis

Fourier Transform Infrared Spectroscopy (FTIR) was used to characterize the pigmented LLDPE blends. Samples with 10 × 10 mm dimensions were cut from the 150 μm thick sheets. Measurements were obtained using an FTIR spectrometer (Nicolet iS50, Thermo Scientific, Waltham, MA, USA). Data were collected across the absorption spectrum from 5 μm to 25 μm. The spectra for the pure and pigmented LLDPE sheets were analyzed with Origin pro 2022 (OriginLab, Northampton, MA, USA) software. Measurements were taken at two different spots on each sheet.

##### Infrared Radiation Analysis

A comprehensive infrared radiation (IR) analysis was conducted to examine if the pigmented LLDPE blends retained IR properties similar to pure LLDPE. We assessed two IR properties—transparency and emissivity—using custom experimental setups. For IR transparency, measurements were obtained using both direct and reflected heat sources to provide a comprehensive understanding of the transparency characteristics. This dual approach was employed because transparency to IR radiation from a direct source may not fully capture a material’s behavior in real-world applications, where IR radiation often reaches surfaces indirectly, such as in shaded environments or when IR radiation is reflected off surrounding surfaces. An IR-opaque fixture was 3D-printed to hold 1 × 1-inch pigmented LLDPE sheets, and data were captured using a FLIR E75 thermal camera (Teledyne FLIR, Wilsonville, OR, USA).

**Figure 2:**
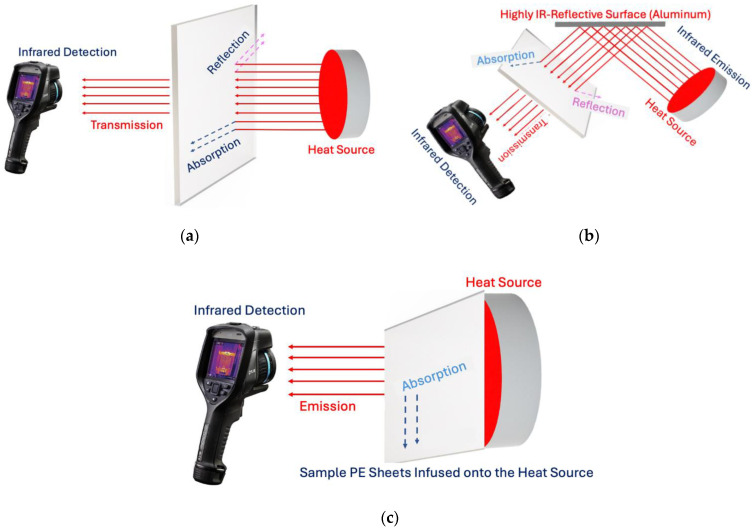
A schematic diagram of the test setup for assessing IR transmittance and emittance. (**a**) The direct heat source setup: The pigmented LLDPE sheets were mounted 1 inch in front of a 49 °C heat source. The IR camera was positioned 2 feet away to detect IR radiation passing through the sheets. (**b**) The reflected heat source setup: A metalized Mylar sheet was used to reflect IR radiation from a 49 °C heat source. The pigmented LLDPE sheets were mounted 1 inch in front of the metalized Mylar sheet at a 45° angle, and the IR camera was positioned 2 feet away at a similar angle to detect the reflected IR radiation passing through the pigmented LLDPE sheets placed in front of the mirror. (**c**) The IR emittance setup: The pigmented LLDPE sheets were placed directly onto a 49 °C heat source. After stabilization, the IR camera detected the radiation emitted from the sheets.

For transparency testing using a direct heat source, the sheets were mounted in the fixture and placed 1 inch in front of a 49 °C heat source. The IR camera, positioned 2 feet away, detected IR radiation passing through the sheets to measure the transparency of each specimen. This principle is illustrated in Figure 2a.

For transparency testing using a reflected heat source, a 1.5 × 1.5-foot metalized Mylar sheet was used as a mirror to reflect the IR radiation. It was placed 2 feet from the heat source at a 45° angle and adjusted until the heat source was fully reflected, as seen by the IR camera positioned at the matching angle. The pigmented LLDPE sheets were placed 1 inch in front of the Mylar sheet, and the IR camera captured the reflected IR radiation to assess the sheets’ transparency to IR from a reflected source. This principle is shown in Figure 2b.

For the emittance tests, the pigmented LLDPE sheets were placed on the 49 °C heat source, and readings were taken after five minutes to allow the sheets to reach the same temperature. Once stable, the emittance radiation was measured using the IR camera positioned 2 feet away, and the IR emission data were recorded. This principle is shown in Figure 2c. Actual images of the setups are shown in Figure S4 (in the Supporting Information).

The IR camera was aimed at three spots on each sheet, and the tests were conducted on two sets of sheets for each property. The average of the readings was recorded as the temperature for each sheet. After capturing the temperature readings, we calculated the IR transmittance and emittance percentages. For IR transmittance, the recorded temperature values were compared with the heat source temperature to determine the percentage of IR radiation passing through each sample using the following formula:

$$\mathrm{IR}\;\mathrm{Transmittance}\;(\%)=\frac{Temperature\;of\;Pigmented\;Sample}{Temperature\;of\;Heat\;Source}\times 100$$

Similarly, the IR emittance was calculated by comparing the temperature readings with the heat source temperature.

##### Ultraviolet–Visible Spectroscopy Analysis

The absorbance of the extruded white-pigmented sheets produced using the ZnS, ZnO, and TiO_2_ pigments was measured using an ultraviolet–visible (UV–VIS) spectrometer (GENESYS ^TM^ 10, Thermo Scientific, Madison, WI, USA), as visual inspection revealed that all colored pigmented LLDPE sheets were clearly opaque but determining the opacity of the white pigments was more difficult. It was compared to the opacity of pure LLDPE. The light beam and detector diameters were approximately 3 mm, with a 6 mm gap between the sheet and the detector. Measurements were taken at two spots on each sheet.

## 3. Results

### 3.1. Mechanical Performance

The tensile strength, Young’s modulus, and elongation at break values of the pigmented blended LLDPE filaments were measured for each blend and compared to pure LLDPE to assess the impact of the pigments on the filaments’ mechanical performance.

**Figure 3:**
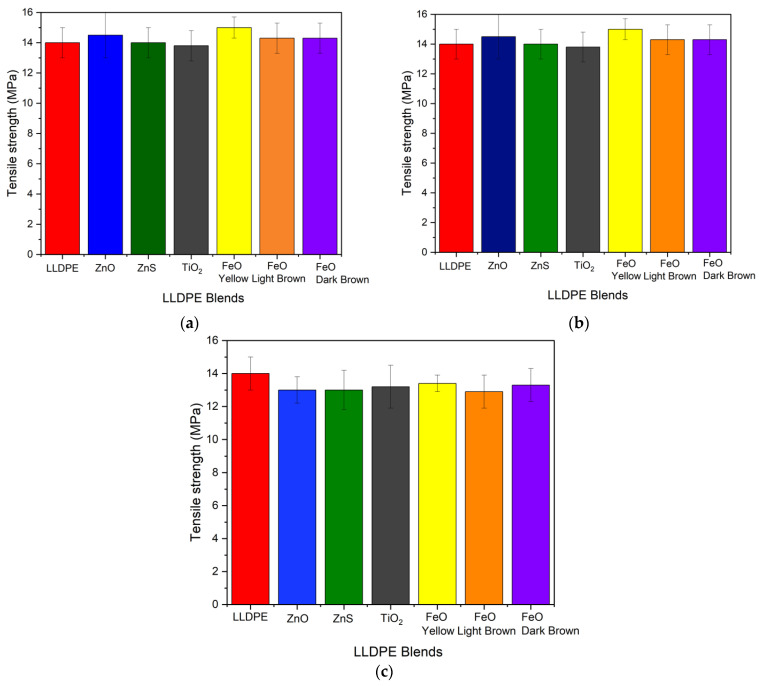
Tensile test results: (**a**) 1 wt% pigmented LLDPE blends, (**b**) 3 wt% pigmented blends, and (**c**) 5 wt% pigmented blends.

Pure LLDPE filaments (1.75 mm diameter) exhibited a high tensile strength of 14 ± 1 MPa (Figure 3). Previous research has reported lower average tensile strength values for pure LLDPE filaments, e.g., 6.1 MPa [39] and 9.9 MPa [40]. The difference between these values and the results of this study can likely be explained by differences in the LLDPE grade, extrusion procedure, filament diameter, testing machine gauge, and other test conditions.

The tensile strength of the filaments containing 1 wt% pigment/wax blends ranged between 13.8 and 15 MPa (Figure 3a), indicating that low concentrations of pigment/wax had a minimal impact on tensile strength. However, as the concentration of pigment/wax increased, a slight decline in tensile strength was observed. For example, filaments with 3 wt% pigment/wax blends exhibited tensile strength values between 12.9 and 13.4 MPa (Figure 3b), while those with 5 wt% blends showed values between 12.0 and 12.5 MPa (Figure 3c).

This reduction in strength at the higher loading rates can be attributed in part to the presence of the polyethylene wax, which is not as strong as LLDPE [41]. These findings are consistent with the existing literature, where researchers reported that higher concentrations of polyethylene wax blended with LDPE led to reductions in mechanical properties [42]. This loss of performance was attributed to crystal phase separation in the blended polyethylene wax/LDPE. Additionally, the shorter molecules of the wax may have facilitated faster flow due to reduced viscosity, ultimately lowering the tensile strength of the blended LLDPE filaments in this study.

Additionally, increased concentrations of filler in composite structures often result in decreases in mechanical performance, including strength [43]. Reasons for this strength decline include filler particle agglomeration, inadequate interfacial adhesion between the filler and the matrix, and larger numbers of stress concentration sites [44]. The combined effect of these factors, along with the lower strength of the wax and the reduced viscosity of the polymer blend, most likely contributed to the observed decline in the tensile strength of the blended LLDPE filaments with higher polyethylene wax/pigment concentrations.

A similar trend was observed for Young’s modulus, as detailed in Table 2. At 1 wt%, all pigmented LLDPE filaments maintained Young’s modulus values similar to that measured for pure LLDPE filaments. The elongation at break values also remained high, ranging from 1150% to 1240%, comparable to that of pure LLDPE at 1200%. This indicates that at low concentrations, the pigments/wax had little effect on both the stiffness and ductility of the material, similar to the tensile strength data.

At 3 wt% pigment/wax loading, a moderate reduction in the Young’s modulus of approximately 20% compared to pure LLDPE was observed across all pigment types. On the other hand, the reduction in the elongation at break was not significant, and the elongation values remained above 1150%.

However, at 5 wt%, a more substantial decrease in the Young’s modulus was seen across all pigments, with values ranging from 90 MPa to 99 MPa. This reduction in stiffness, combined with the lower tensile strength (12.0–12.5 MPa), indicates that the addition of higher concentrations of pigments and wax weakened the overall mechanical performance of the filament. The elongation at break values also decreased, falling below 1100%, leading to a moderate reduction in ductility. However, the material retained a relatively good strain capacity.

The observed trends in the mechanical properties with increasing pigment/wax concentrations were consistent with the general behavior of filled polymer systems. As noted by Fu et al. in their comprehensive review, the addition of rigid particles to a polymer matrix can lead to increased stiffness but decreased ductility, with this effect becoming more pronounced at higher filler loadings [45]. The retention of high elongation values even at 5 wt% loading suggests that the LLDPE matrix still dominated the overall mechanical behavior, which is characteristic of thermoplastic elastomers like LLDPE [46].

### 3.2. IR Transmission Properties

#### 3.2.1. FTIR Spectroscopy Analysis

**Figure 4:**
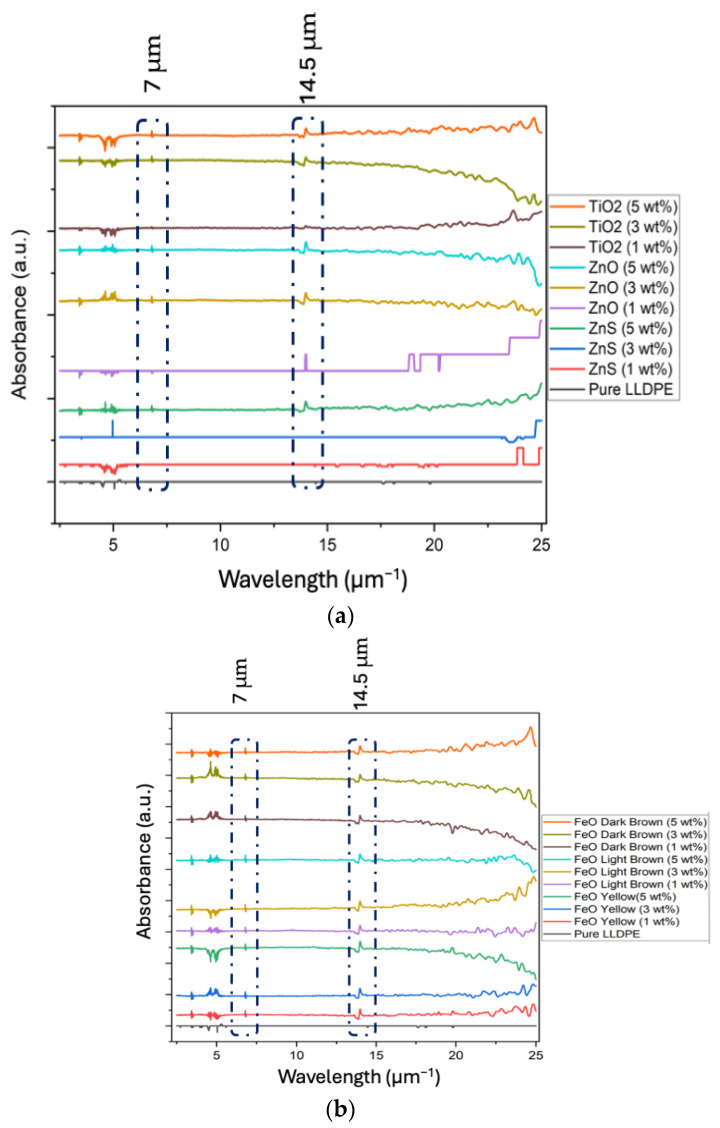
FTIR spectra for pure LLDPE and 1, 3, and 5 wt% pigmented LLDPE: (**a**) white-pigmented LLDPE blends; (**b**) colored pigmented LLDPE blends.

FTIR spectra were collected for sheets molded from the pure and filled LLDPE extruded filaments at 1, 3, and 5 wt% loading concentrations. The objective was to assess whether the addition of pigments affected LLDPE’s IR properties, particularly in the 8–12 μm range, which corresponds to the human body’s IR spectrum [47]. Figure 4a shows the results for the white pigments (ZnS, ZnO, and TiO_2_), and Figure 4b shows the results for the colored pigments (FeO yellow, light brown, and dark brown).

As shown in Figure 4a,b, regardless of the pigment color, two new peaks appeared at approximately 7 and 14.5 μm in the pigmented samples compared to the pure LLDPE sample. These peaks can be attributed to CH_2_ bending vibration and are consistent with findings in the literature [48,49]. This may be due to the blending process, particularly with PE wax, which can facilitate side reactions and structural rearrangements in the polymer composite, such as the formation of methylene (CH_2_) groups through partial decomposition, oxidation, or thermal degradation of the wax or pigment materials during the manufacturing processes [50]. In addition, the PE wax itself may inherently contain C-CH_2_ groups, contributing to these new peaks when mixed with LLDPE [48]. However, no peaks were observed in the 8–12 μm range for the pigmented LLDPE blends, similar to pure LLDPE. This result is attributed to the choice of the pigments used in this study, which were selected for their IR transparency.

#### 3.2.2. IR Transparency

IR transparency tests were conducted with a direct heat source for pure LLDPE sheets as well as for all pigment-blended sheet samples at varying concentrations and thicknesses. Data were collected using an IR camera, which captured both the thermal images and temperature readings of the polymer sheets positioned in front of a heat source.

**Figure 5:**
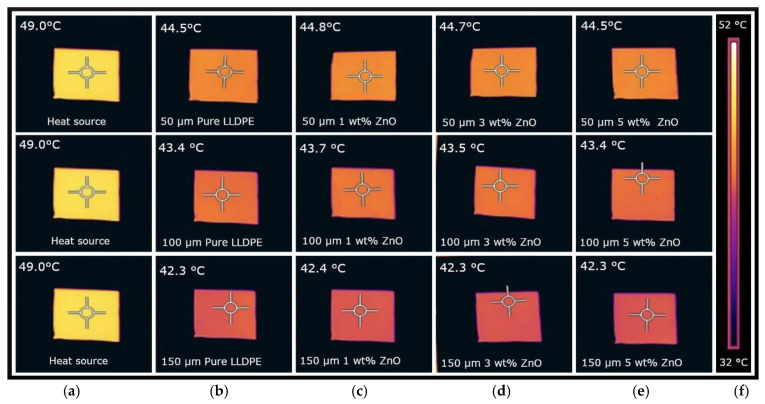
Results of IR transparency from direct heat source measurements for pure and ZnO-pigmented LLDPE with 1, 3, and 5 wt% concentrations: (**a**) heat source, (**b**) pure LLDPE, (**c**) 1 wt%, (**d**) 3 wt%, (**e**) 5 wt%, and (**f**) temperature scale.

As shown in Figure 5b, the pure LLDPE sheets exhibited temperatures of 44.5 °C, 43.5 °C, and 42.3 °C for sheet thicknesses of 50, 100, and 150 μm, respectively, while the heat source temperature was 49 °C. This suggests that pure LLDPE transmits more than 85% of IR radiation, indicating a high level of IR transparency.

**Figure 6:**
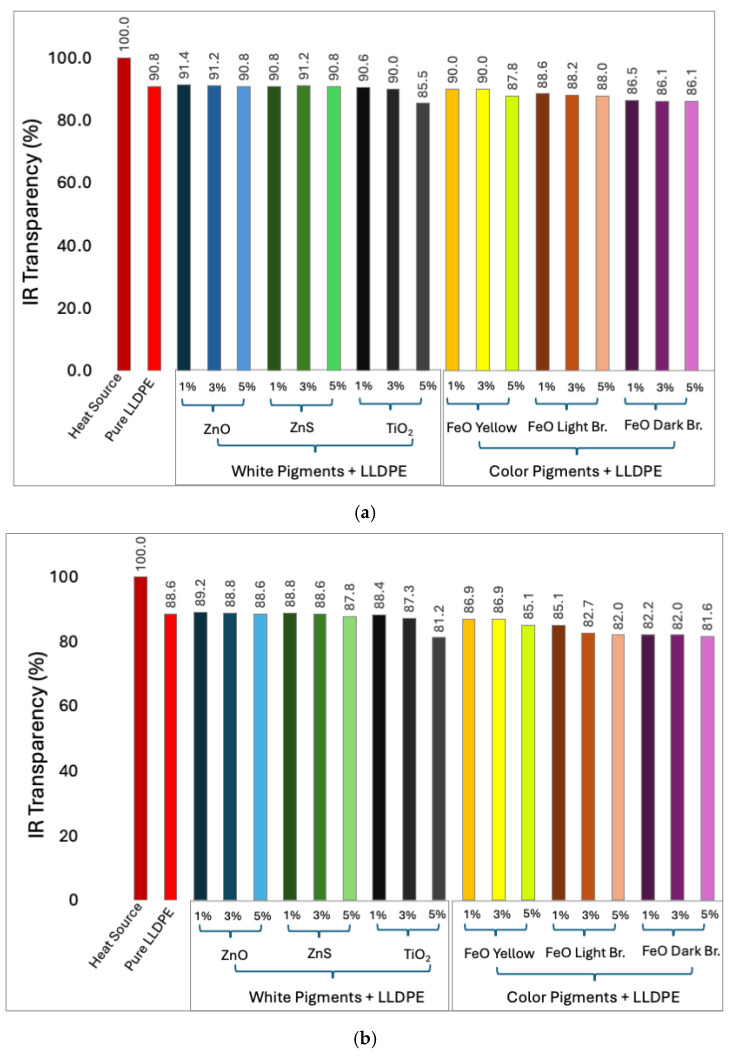

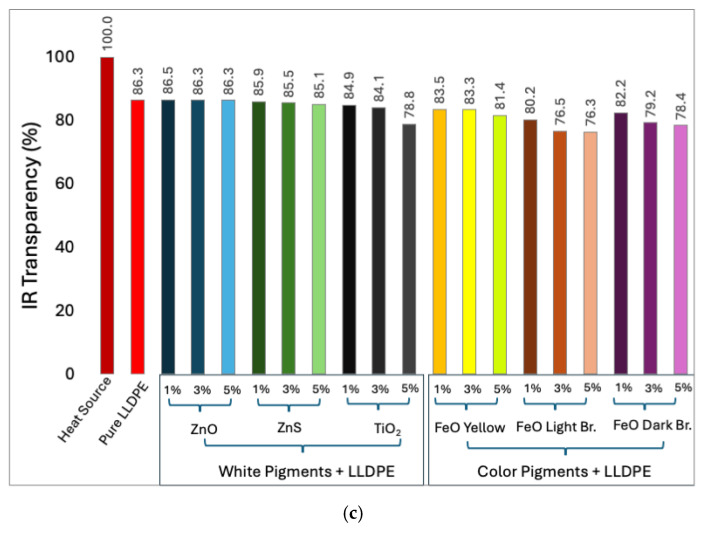
IR transparency from direct heat source for pure LLDPE sheets and pigmented sheets with white pigments (ZnO, ZnS, and TiO_2_) and colored pigments (FeO yellow, light brown, and dark brown) at 1, 3, and 5 wt% concentrations with sheet thicknesses of (**a**) 50, (**b**) 100, and (**c**) 150 μm.

Figure 5c–e also show thermal images recorded for the ZnO-pigmented sheets at different thicknesses and loading concentrations. The IR transparency results for the white-pigmented LLDPE sheets indicate consistent behavior across the different pigments (Figure 6). The ZnO and ZnS blends exhibited similar transparency levels, approximately 91% (Figure 6a), 89% (Figure 6b), and 86% (Figure 6c) for the 50, 100, and 150 µm thick specimens, respectively. The TiO_2_ blends with higher loading concentrations (5 wt%) demonstrated slightly lower IR transparency, with values around 85.5% (Figure 6a), 81.2% (Figure 6b), and 78.8% (Figure 6c) for the 50 µm, 100 µm, and 150 µm specimens, respectively.

Among the color-pigmented LLDPE blends, the FeO yellow-pigmented LLDPE exhibited slightly better IR transparency, ranging from 81% to 90% across various concentrations and sheet thicknesses (Figure 6). In comparison, FeO light brown and FeO dark brown showed slightly lower IR transparency levels between 76% and 89%. The overall order of decreasing IR transparency across all pigments was as follows: ZnO/ZnS, TiO_2_, FeO yellow, FeO light brown, and FeO dark brown. This finding is consistent with the well-documented optical properties of these materials. ZnO’s wide bandgap and minimal absorption in the infrared region [51] allow it to transmit a higher percentage of IR radiation compared to TiO_2_, which has higher absorption due to its scattering properties. Additionally, the FeO yellow, light brown, and dark brown pigments contain Fe_3_O_4_, which is known for its strong IR absorption [52]. This explains the lower transparency observed in the FeO-pigmented sheets, particularly those pigmented with FeO dark brown.

It is also possible to observe in Figure 6 that the IR transparency generally decreased as the pigment concentration increased. The larger number of pigment particles led to higher IR radiation absorption and scattering and prevented its transmission through the material. Additionally, higher pigment concentrations increased the likelihood of particle aggregation, forming larger clusters that further scattered and absorbed IR radiation.

The IR transparency also slightly decreased with increasing sheet thickness. In thinner sheets, the IR radiation penetrated more readily because it encountered less matter with IR-absorbing potential. As the thickness increased, the likelihood of IR radiation interacting with IR-absorbing groups rose, leading to greater IR absorption and a reduction in IR transparency.

**Figure 7:**
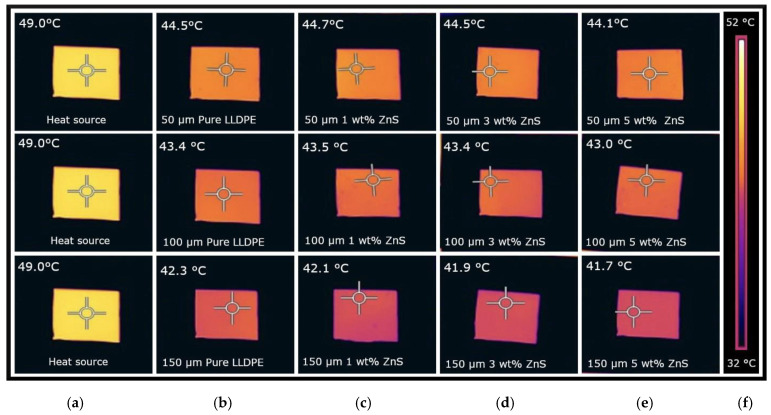
Results of IR transparency from reflected heat source for pure and ZnS-pigmented LLDPE with 1, 3, and 5 wt% concentrations: (**a**) heat source, (**b**) pure LLDPE, (**c**) 1 wt%, (**d**) 3 wt%, (**e**) 5 wt%, and (**f**) temperature scale.

Examples of thermal images measuring the IR transmittance from a reflected heat source for 50, 100, and 150 μm pure LLDPE and ZnS-pigmented sheets at 1, 3, and 5% concentrations are shown in Figure 7. Across all samples, a comparatively lower temperature was observed with a higher sheet thickness, indicating reduced IR transmittance.

**Figure 8:**
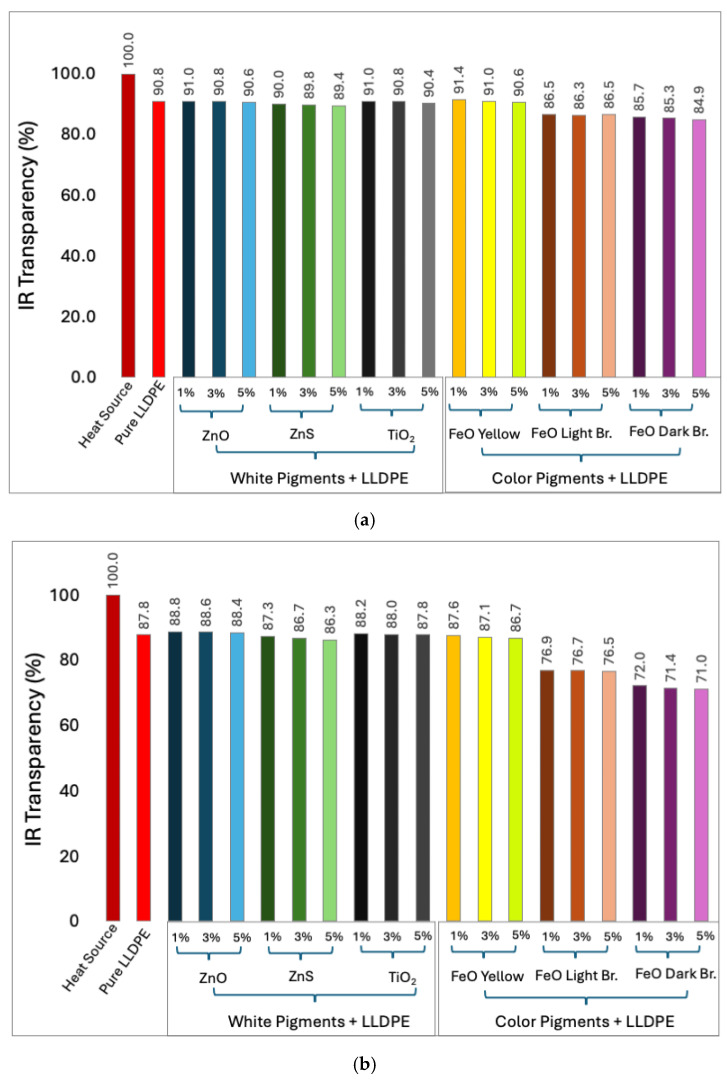

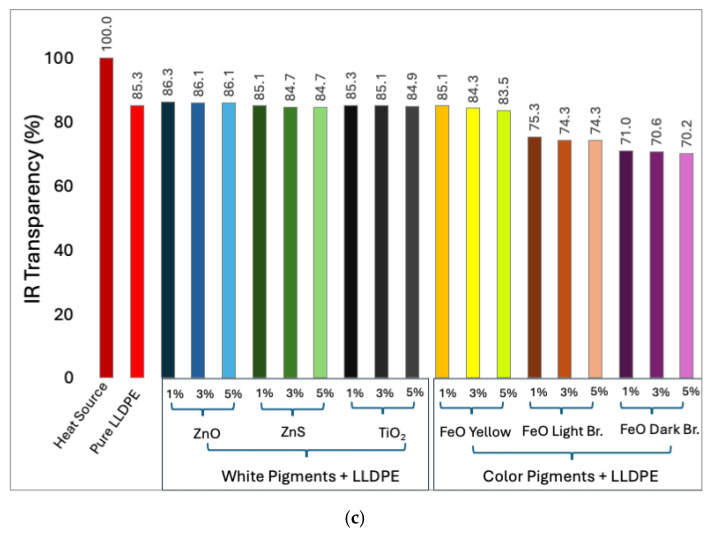
IR transparency from reflected heat source for pure LLDPE sheets and pigmented sheets with white pigments (ZnO, ZnS, and TiO_2_) and colored pigments (FeO yellow, light brown, and dark brown) at 1, 3, and 5 wt% concentrations with sheet thicknesses of (**a**) 50, (**b**) 100, and (**c**) 150 μm.

The IR transmittance results from the reflected heat source for pure and pigment-blended LLDPE sheets at various thicknesses and concentrations are presented in Figure 8. For the white pigments (ZnO, ZnS, and TiO_2_), the effect on the IR transmittance from the reflected heat source was minimal across all three thicknesses tested. Specifically, the IR transmittance values were approximately 90% (Figure 8a), 87% (Figure 8b), and 85% (Figure 8c) for the 50, 100, and 150 μm white-pigmented LLDPE sheets, respectively.

Among the colored pigments, the FeO yellow-pigmented LLDPE showed higher IR transmittance from the reflected heat source compared to the other colored pigments. For example, the FeO yellow-pigmented LLDPE exhibited IR transmittance values of 91%, 87%, and 84% for the 50, 100, and 150 μm sheets, respectively. In contrast, the FeO light brown-pigmented sheets demonstrated IR transmittance values between 74 and 86%, while the FeO dark brown-pigmented sheets exhibited values ranging from 70 to 85% across the different concentrations and thicknesses.

As shown in Figure 7, the ZnS-pigmented LLDPE exhibited minimal variation in IR reflectance with changes in sheet thickness and pigment concentration. However, the overall trend became less conclusive when comparing the IR transmittance results from the reflected heat source for all white-pigmented LLDPE sheets (Figure 8). In contrast, the color-pigmented LLDPE sheets showed pigment type, concentration, and sheet thickness effects. A consistent decrease in temperature was observed across the color-pigmented samples as the pigment concentration and thickness increased.

The IR transmittance results from both the direct and reflected heat sources consistently demonstrated the high IR transparency of the pure and pigmented LLDPE sheets across the various pigments, concentrations, and thicknesses. For the white pigments (ZnO, ZnS, and TiO_2_), the results suggest that minimal variations in IR transparency occurred, with transmittance values remaining above 85% across both the direct and reflected heat source tests. This indicates that white pigments, even at higher concentrations, do not significantly alter the IR transparency of LLDPE. This finding implies they are suitable for applications where IR transparency is desired alongside visible opacity.

In contrast, the color-pigmented LLDPE blends showed slight reductions in IR transparency, particularly for the FeO light brown and FeO dark brown pigments, which demonstrated transmittance levels as low as 70% for the thicker and higher-concentration samples. This trend was consistently observed in both transmittance tests.

The overall IR transmittance values from the reflected heat source were lower than the IR transparency values from the direct heat source due to the initial reflection of the radiation by the metalized Mylar sheet, which introduced additional absorption and scattering before reaching the LLDPE sheets. Despite this, the pigmented LLDPE sheets with lower thicknesses and pigment concentrations demonstrated transmittance values comparable to those of pure LLDPE.

#### 3.2.3. IR Emittance

The IR emittance results of the pure and pigment-blended LLDPE sheets are shown in Figures S5 and S6 (in the Supporting Information). All samples, regardless of thickness, pigment type, and loading concentration, reached the same temperature as the heat source (49 °C). This demonstrates that both pure LLDPE and pigmented blends have similar IR emittance properties.

### 3.3. Visible Opacity Performance

**Figure 9:**
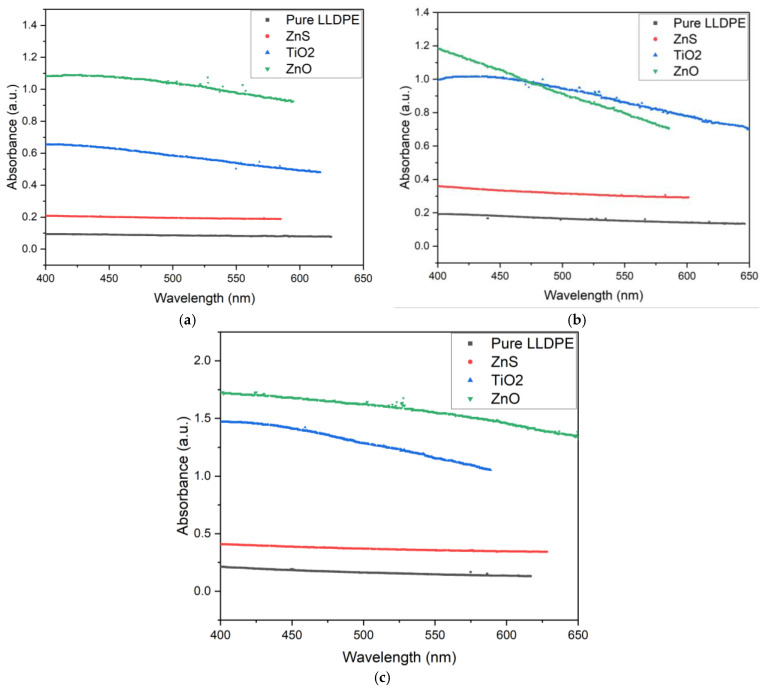
UV–VIS spectra for white-pigmented LLDPE blends at 1 wt% loading for (**a**) 50, (**b**) 100, and (**c**) 150 μm thick sheets.

**Figure 10:**
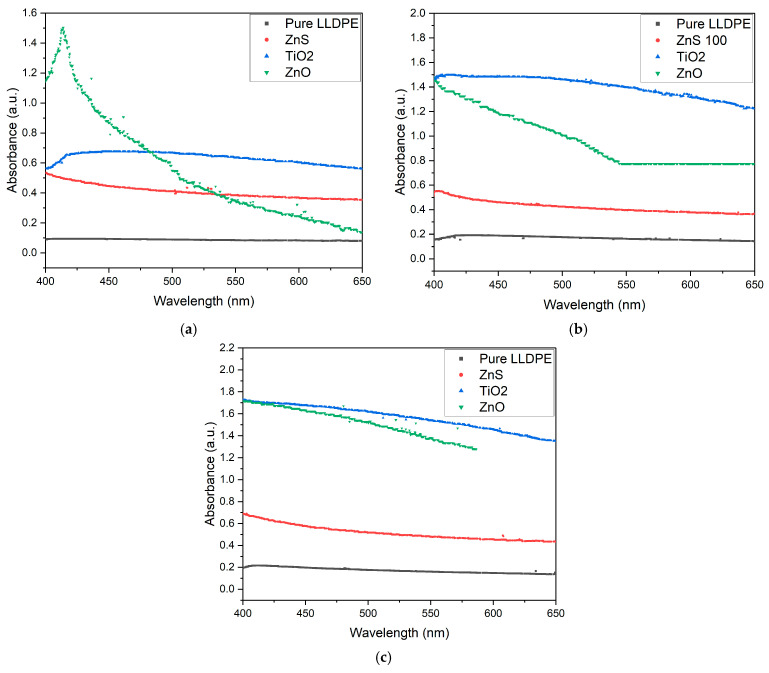
UV–VIS spectra for white-pigmented LLDPE blends at 3 wt% loading for (**a**) 50, (**b**) 100, and (**c**) 150 μm thick sheets.

**Figure 11:**
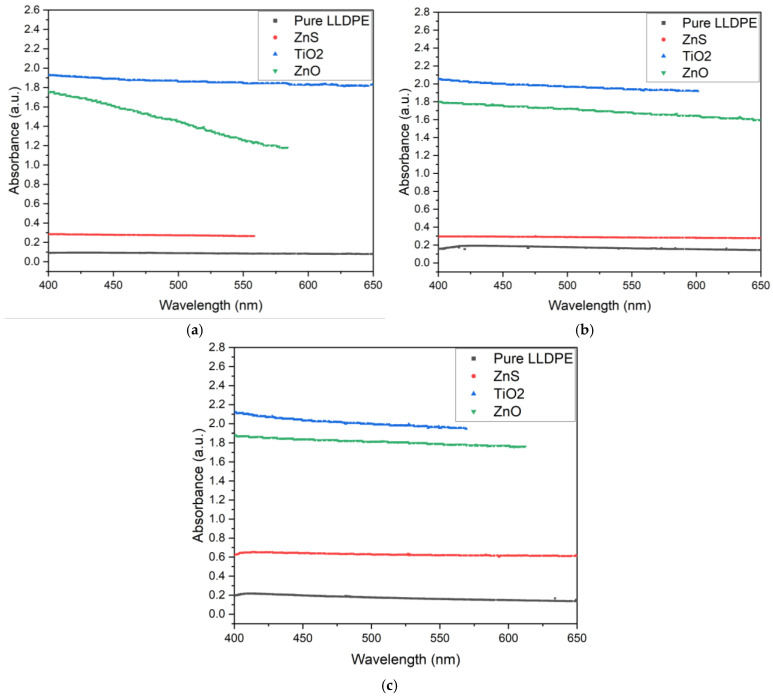
UV–VIS spectra for white-pigmented LLDPE blends at 5 wt% loading for (**a**) 50, (**b**) 100, and (**c**) 150 μm thick sheets.

The UV–VIS spectra in the visible-light range (400–650 nm) recorded for 50, 100, and 150 μm thick sheets with 1, 3, and 5 wt% loading concentrations using the white pigments (ZnS, ZnO, and TiO_2_) are shown in Figure 9, Figure 10 and Figure 11 in comparison with pure LLDPE. The pure LLDPE sheets showed very low absorbance (0.1–0.25 depending on the sheet thickness), indicating high visible-light transparency, particularly at higher wavelengths. This high transparency of LLDPE for visible light can be attributed to the linear structure of the molecule, which involves short-chain branching and low crystallinity [53]. This visible spectrum can be influenced by various factors including the polymer composition, additives, processing conditions, and the thickness of the sample [54,55]. This explains the increase in absorbance observed as the sheet thickness increased.

As shown in Figure 9, Figure 10 and Figure 11, the white pigment-blended LLDPE sheets (ZnS, TiO_2_, and ZnO) absorbed more light than the pure LLDPE. All pigments exhibited decreasing absorbance as the wavelength increased, consistent with the behavior observed with pure LLDPE. Two key trends were observed: (1) The absorbance increased with the sheet thickness. (2) The absorbance also rose with the pigment concentration. In both cases, this behavior can be attributed to increased scattering and absorption of light by the larger quantities of pigments in the sheets [56].

However, the ZnO-pigmented LLDPE sheets exhibited a slightly different behavior compared to the ZnS, TiO_2_, and pure LLDPE sheets, whose visible-light absorbance varied in a consistent manner across the sheet thicknesses and pigment concentrations. For example, a steeper decrease in absorbance was observed as a function of the wavelength for the 100 μm thick ZnO-pigmented LLDPE sheets compared to the 50 μm and 150 μm sheets (Figure 9). Additionally, the absorbance for the ZnO-loaded LLDPE sheets at the 3 and 5 wt% concentrations were lower than for the TiO_2_ sheets (Figure 10 and Figure 11), while the reverse was observed at the 1 wt% loading concentration (Figure 9). Finally, the absorbance variation as a function of the wavelength exhibited non-monotonic behavior at the 3 wt% loading concentration for the 50 μm thick sheets with ZnO nanoparticles.

This difference in behavior for the ZnO-loaded sheets could be attributed to several factors. First, the dispersion and distribution of the ZnO particles within the LLDPE matrix may vary for different thicknesses, potentially causing non-uniform light scattering [53]. This would result in localized changes in absorbance at specific thicknesses. Second, there may be optical interference effects, such as constructive or destructive interference between light waves interacting with the ZnO particles at certain sheet thicknesses, which could explain the reduced absorbance at 100 μm [51]. The fact that at higher concentrations (3 wt% and 5 wt%) the TiO_2_-loaded sheets surpassed the ZnO-loaded sheets in absorbance (Figure 10 and Figure 11) shows the interplay between the pigment concentration and the sheet thickness in determining the overall optical properties of the LLDPE/pigment blended polymer sheets.

### 3.4. Effect of Surface Micro-Texturing

**Figure 12:**
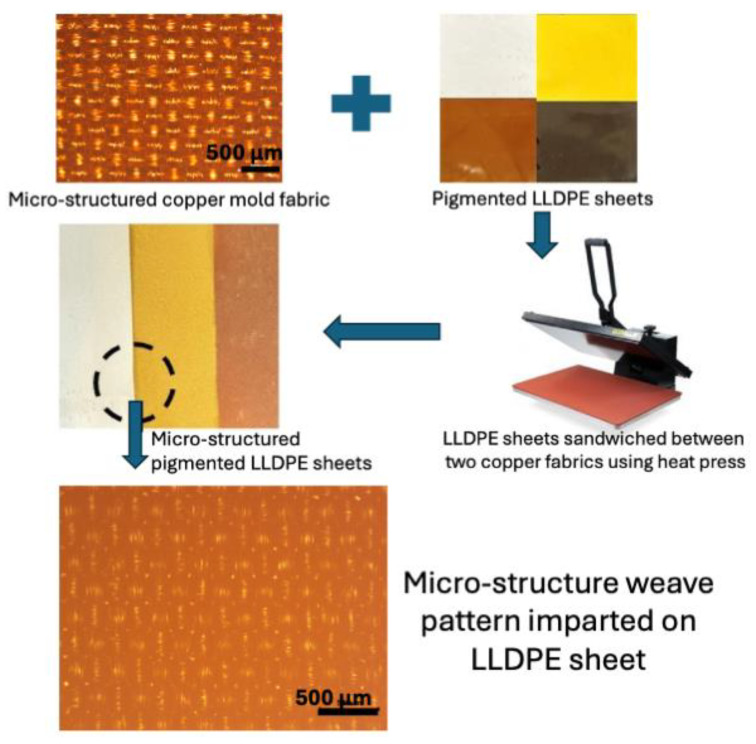
A schematic diagram of the protocol for micro-structuring the pigmented LLDPE sheets.

An attempt was made to further tune the IR transparency of the pigmented LLDPE sheets by micro-structuring the sheet surfaces. The LLDPE sheets were micro-structured by sandwiching them between two micro-structured copper cloths, followed by heat-pressing at temperatures near the melting point of LLDPE for one minute (Figure 12). Upon cooling, the sheets were examined under a microscope, which confirmed that the micro-structures from the copper cloths had successfully transferred to the LLDPE sheets (Figure 12).

**Figure 13:**
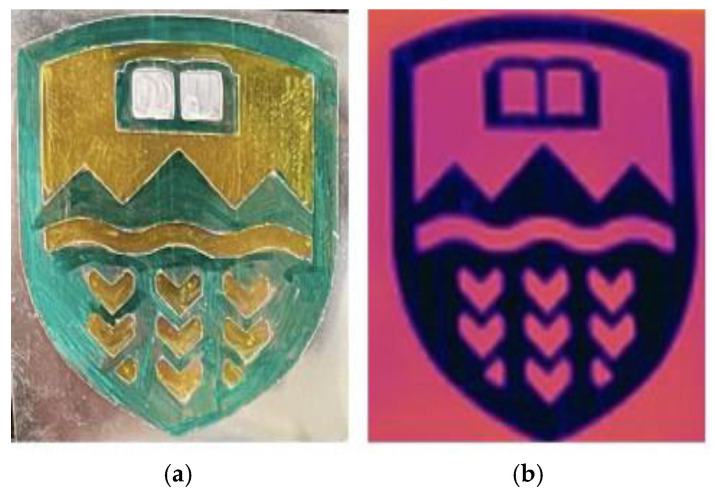
The University of Alberta logo made of metalized Mylar under visible light (**a**) and when located 1 inch from a heat source and viewed with an IR camera (**b**).

The micro-structured LLDPE sheets were tested for IR transparency against a 49 °C heat source. The University of Alberta logo was produced using Mylar and metalized Mylar (Figure 13a). When placed 1 inch from a heat source and viewed with an IR camera, the logo revealed areas of low and high IR emission (Figure 13b): the polymer areas appeared red, as Mylar highly emits IR, while the metallized Mylar showed as blue, as the metallization has low emission and primarily reflects the ambient temperature.

**Figure 14:**
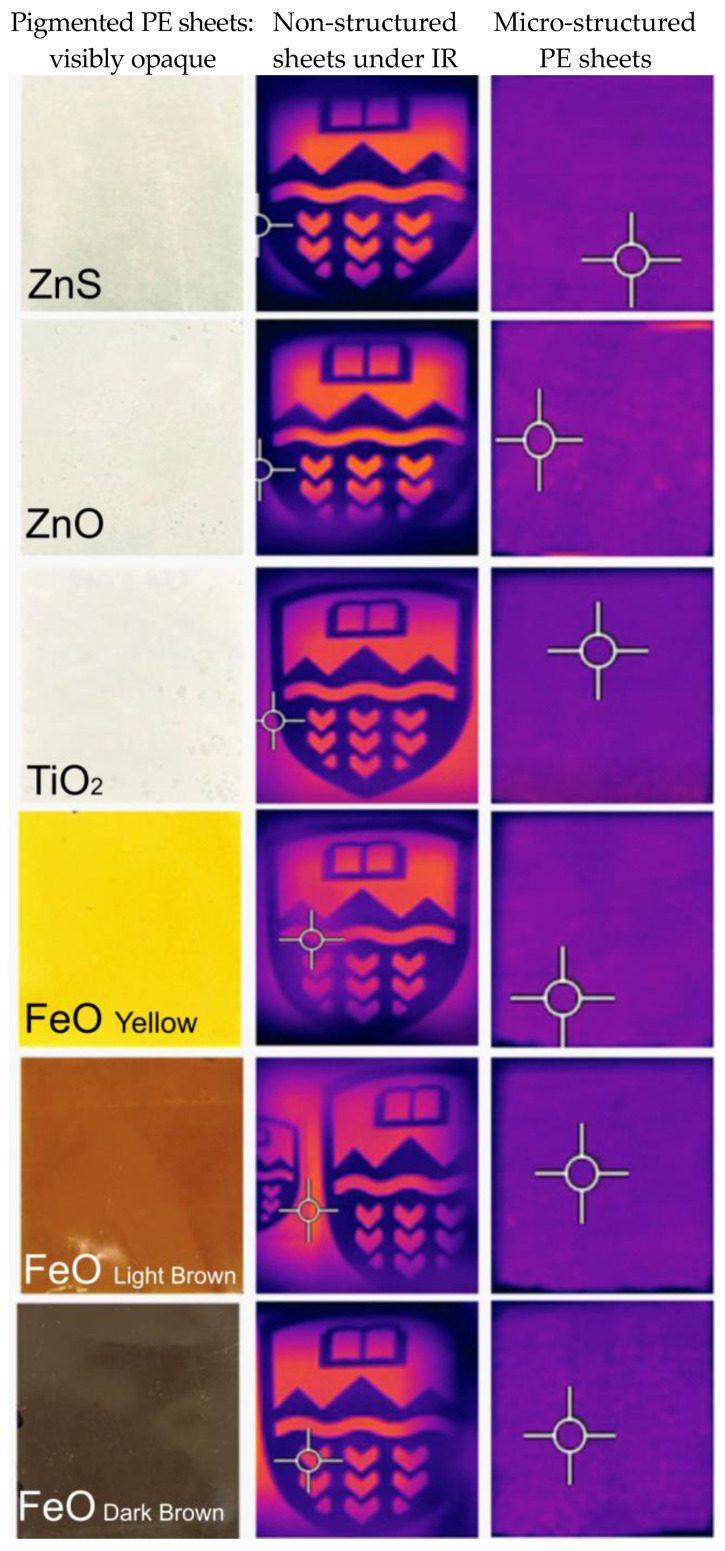
Effects of micro-structuring of pigmented PE sheets on IR transparency.

Next, the pigmented LLDPE sheets characterized in Section 3.2 and Section 3.3 were positioned 2 mm in front of the logo and viewed using the IR camera (Figure 14). The logo remained clearly visible, in agreement with the high IR transparency of the pigmented LLDPE sheets shown in Section 3.2. On the other hand, when the micro-structured pigmented LLDPE sheets were placed in front of the logo, they completely obscured the logo. This can be attributed to the scattering of IR radiation that occurred in the micro-structured sheets [57]. Since the micro-structures were in the 1–10 micrometer range, they were very efficient at scattering IR radiation in the mid-infrared region. This simple technique therefore shows great potential for creating polymer sheets with tunable IR properties, shifting from transparent to scattering based on application needs.

## 4. Discussion

In this study, we present a comprehensive investigation of the effects of pigment incorporation into LLDPE sheets, focusing on their mechanical properties, IR transparency, and visible opacity. The findings collectively provide valuable insights into how this material’s optical and mechanical performance can be tuned to meet the specific needs of thermal management and other relevant applications.

The mechanical performance of the pigmented LLDPE sheets showed minimal changes at lower pigment concentrations, as they exhibited tensile strength and flexibility values similar to pure LLDPE (Figure 3 and Table 2). However, as the pigment concentration increased, slight declines in the tensile strength and Young’s modulus were observed, especially at the highest pigment concentration (5 wt%). This change can be attributed to the reduced structural integrity caused by the incorporation of polyethylene wax, which aids pigment dispersion but has lower strength compared to LLDPE [41]. Despite this, the material retained good ductility, especially at concentrations up to 3 wt%, which is advantageous for many applications that require a balance between mechanical strength and flexibility [58].

The IR transparency of the LLDPE sheets was largely unaffected by pigment addition, particularly in the range of 8–12 μm (Figure 4), which is critical for radiative cooling applications. However, the impact of an increasing pigment concentration on IR transparency was evident, especially with the colored pigments, such as FeO yellow and FeO dark brown (Figure 6), where slight reductions in transparency were observed. This trend highlights the increased scattering and absorption effects at higher pigment concentrations.

The increase in visible opacity with the pigment concentration and sheet thickness directly correlated with the observed reduction in visible-light transmittance (Figure 9, Figure 10 and Figure 11). This behavior was more pronounced with the increases in pigment concentration and sheet thickness. As the pigment concentration increases, there are more particles available to scatter and absorb light, leading to increased opacity [59]. Similarly, as the sheet thickness increases, light passes through more pigment particles, resulting in greater absorption and scattering [60]. The combined effect of increasing both the pigment concentration and the sheet thickness leads to a multiplicative impact on opacity, as the amount of pigment interacting with light increases in both dimensions.

The micro-structuring of the LLDPE sheets introduced a novel method for enhancing IR scattering. The micro-structured sheets, when placed in front of the IR camera, effectively blocked the underlying logo, indicating substantial scattering of IR radiation (Figure 14). This result connects directly to the findings on IR transparency, as it demonstrates that while pigments can influence transmission, the micro-structural design adds an additional layer of control over the material’s thermal properties. This micro-structuring technique provides a way to fine-tune the IR behavior of the material, shifting from transparency to scattering depending on the application needs. The ability to achieve such tunable properties using simple surface modifications suggests significant potential for creating adaptable materials for various thermal management applications.

The primary scientific takeaway from this study is the confirmation that LLDPE sheets can be effectively pigmented to achieve tunable visible opacity while preserving essential IR transparency, with the added benefit of enhancing IR scattering through micro-structuring. These properties are crucial for applications in radiative cooling and energy-efficient designs, where both optical and thermal performance are important. Moreover, the findings suggest that by adjusting the pigment concentrations and using micro-structural modifications, it is possible to customize this material for a wide range of potential uses, from textiles to packaging and construction materials.

Looking forward, further research could focus on optimizing the pigment selection and concentration to enhance this material’s optical properties without sacrificing mechanical strength. Additionally, exploring the durability of these pigmented LLDPE sheets under real-world conditions, such as exposure to UV radiation and physical wear, will be essential to evaluate their long-term performance. Expanding this study to include other polymers with similar compounding techniques could also lead to broader applications across different industries.

## 5. Conclusions

This study successfully developed a simple method to generate custom LLDPE compounds with a variety of pigments while preserving its desirable IR properties. The incorporation of six different pigments (ZnO, TiO_2_, ZnS, FeO yellow, FeO light brown, and FeO dark brown) at varying concentrations (1, 3, and 5 wt%) was shown to result in visually vibrant sheets with high IR transparency. Mechanical testing demonstrated that low concentrations of pigments had minimal impacts on tensile strength and Young’s modulus, while higher concentrations led to slight reductions in mechanical performance, which was consistent with the behavior of a filled polymer matrix.

FTIR analysis confirmed that the addition of the pigments did not significantly alter the IR transparency in the 8–12 μm range, which is crucial for thermal applications. IR testing for transparency and emittance further validated that the pigmented LLDPE sheets retained high IR performance across different thicknesses and pigment concentrations. UV–VIS analysis revealed that visible-light absorbance increased with the pigment concentration and the sheet thickness. Finally, micro-structuring the pigmented LLDPE sheets showed the potential to tune the IR properties based on application needs.

This work demonstrates the potential of pigmenting LLDPE to achieve tunable visible properties without significantly compromising its IR transparency. These findings open new avenues for the use of pigmented LLDPE sheets in applications such as thermal management, radiative cooling, and camouflage, where both visible opacity and IR transparency are critical. Future work could focus on expanding this approach to other grades of polyethylene and further exploring the potential to tune IR properties through micro-structuring techniques.

## Figures and Tables

**Table 1:** List of pigmented LLDPE blends prepared.

Sample Code	Concentration Levels (wt%)		
	Pigment	Polymer Wax	LLDPE
Pure LLDPE	0	0	100
1 wt% ZnO-LLDPE	1	1	98
3 wt% ZnO-LLDPE	3	3	95
5 wt% ZnO-LLDPE	5	5	90
1 wt% ZnS-LLDPE	1	1	98
3 wt% ZnS-LLDPE	3	3	95
5 wt% ZnS-LLDPE	5	5	90
1 wt% TiO_2_-LLDPE	1	1	98
3 wt% TiO_2_-LLDPE	3	3	95
5 wt% TiO_2_-LLDPE	5	5	90
1 wt% FeO Yellow–LLDPE	1	1	98
3 wt% FeO Yellow–LLDPE	3	3	95
5 wt% FeO Yellow–LLDPE	5	5	90
1 wt% FeO Light Brown–LLDPE	1	1	98
3 wt% FeO Light Brown–LLDPE	3	3	95
5 wt% FeO Light Brown–LLDPE	5	5	90
1 wt% FeO Dark Brown–LLDPE	1	1	98
3 wt% FeO Dark Brown–LLDPE	3	3	95
5 wt% FeO Dark Brown–LLDPE	5	5	90

**Table 2:** Young’s modulus and elongation at break values of pure and pigmented LLDPE filaments at 1 wt%, 3 wt%, and 5 wt% pigment/wax concentrations.

	1%		3%		5%	
	Young Modulus (MPa)	Elongation at Break (%)	Young Modulus (MPa)	Elongation at Break (%)	Young Modulus (MPa)	Elongation at Break (%)
Pure LLDPE	145 ± 30	1200 ± 50	145 ± 30	1200 ± 50	145 ± 30	1200 ± 50
ZnS	140 ± 40	1240 ± 30	113 ± 30	1175 ± 35	90 ± 40	1055 ± 40
TiO_2_	134 ± 25	1225 ± 30	115 ± 25	1155 ± 25	99 ± 25	1100 ± 30
ZnO	141 ± 40	1220 ± 25	120 ± 20	1170 ± 30	98.5 ± 30	1080 ± 25
FeO Yellow	143 ± 25	1230 ± 20	122 ± 25	1160 ± 30	95 ± 15	1070 ± 25
FeO Light Brown	146 ± 15	1165 ± 40	110 ± 20	1175 ± 40	90 ± 25	1040 ± 35
FeO Dark Brown	147 ± 20	1150 ± 50	110 ± 30	1190 ± 35	92 ± 35	1035 ± 30

## Data Availability

Data is available from the authors upon reasonable request.

## Supplementary Materials

The following supporting information can be downloaded at: https://www.mdpi.com/article/10.3390/mi16020178/s1, Table S1: Extrusion parameters for filament fabrication; Figure S1: (a) White filaments: ZnS, TiO_2_, ZnO; (b) Colored pigments: FeO yellow, light brown, dark brown; Figure S2: Pigmented PE sheets were formed by hydraulically heat pressing for three different loading concentrations of 1, 3, 5 wt% for each pigment; Figure S3: Pigmented LLDPE sheets with 50, 100, 150 μm thicknesses (vertical lines in the pictures) and 1, 3, 5 wt% loading concentrations (horizontal lines) for six pigments; Figure S4: Setup for IR analysis; Figure S5: FeO yellow IR emissivity test for different sheet thicknesses (50, 100, 150 μm) and different loading concentrations (1, 3, and 5 wt%). Images for the heat source and pure LLDPE sheets at different thicknesses are provided as a comparison; Figure S6: Results of the IR emissivity test.
