# Supplementary material for: Development of Visibly Opaque Polyolefin Sheets While Preserving Infrared-Light Transparency
Source: Micromachines (Basel). 2025 Jan 31;16(2):178. doi: 10.3390/mi16020178 (PMC11857700; doi:10.3390/mi16020178)
Supplement: Supplementary file 1 [file micromachines-16-00178-s001.zip › micromachines-3311009-supplementary.pdf]

Development of Visibly Opaque Polyolefin Sheets While Preserving Infrared Light Transparency

Md. Saiful Hoque[1], Mehnab Ali[1], Ziaoruo Sun[1], Asad Asad[1], Patricia Dolez[2], James Hogan[1], Dan Sameoto[1*]

Department of Mechanical Engineering, University of Alberta, Edmonton, AB, Canada.
Department of Human Ecology, University of Alberta, Edmonton, AB, Canada.

*Corresponding Author: sameoto@ualberta.ca

Table S1: Extrusion parameters for filament fabrication

Extrusion parameters	Value
Extrusion Temperature	190 °C
Speed of rotation	6 RPM
Ambient temperature	22 °C
Extrusion rate	14 inches/min

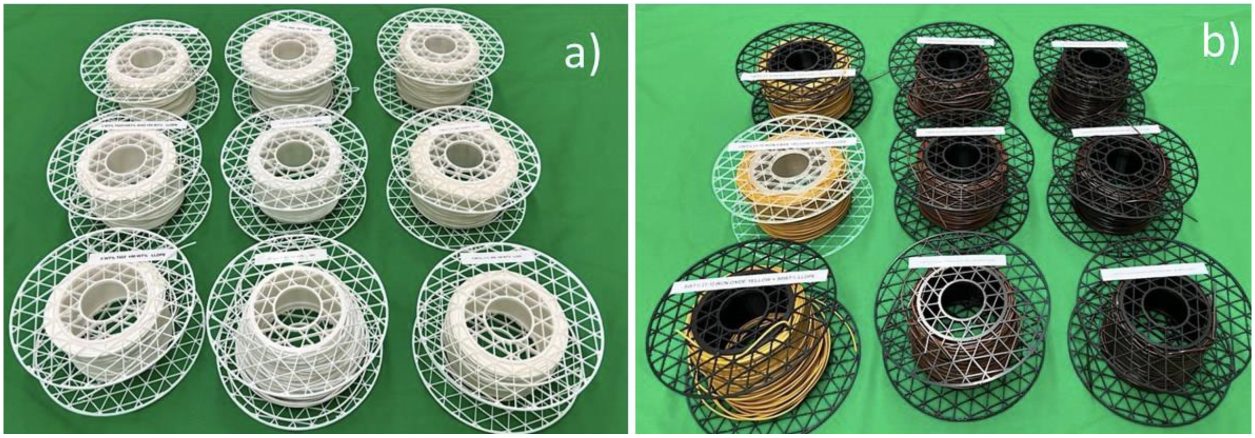

Figure S1: a) White filaments: ZnS, TiO_2, ZnO; b) Colored pigments: FeO yellow, light brown, dark brown.

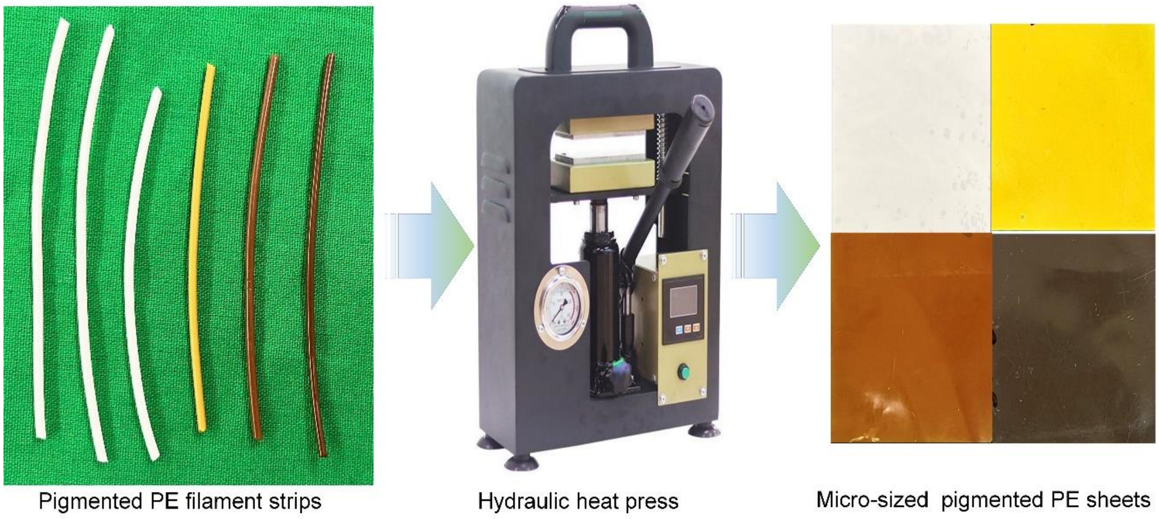

Figure S2: Pigmented PE sheets were formed by hydraulically heat pressing for three different loading concentrations of 1, 3, 5 wt% for each pigment.

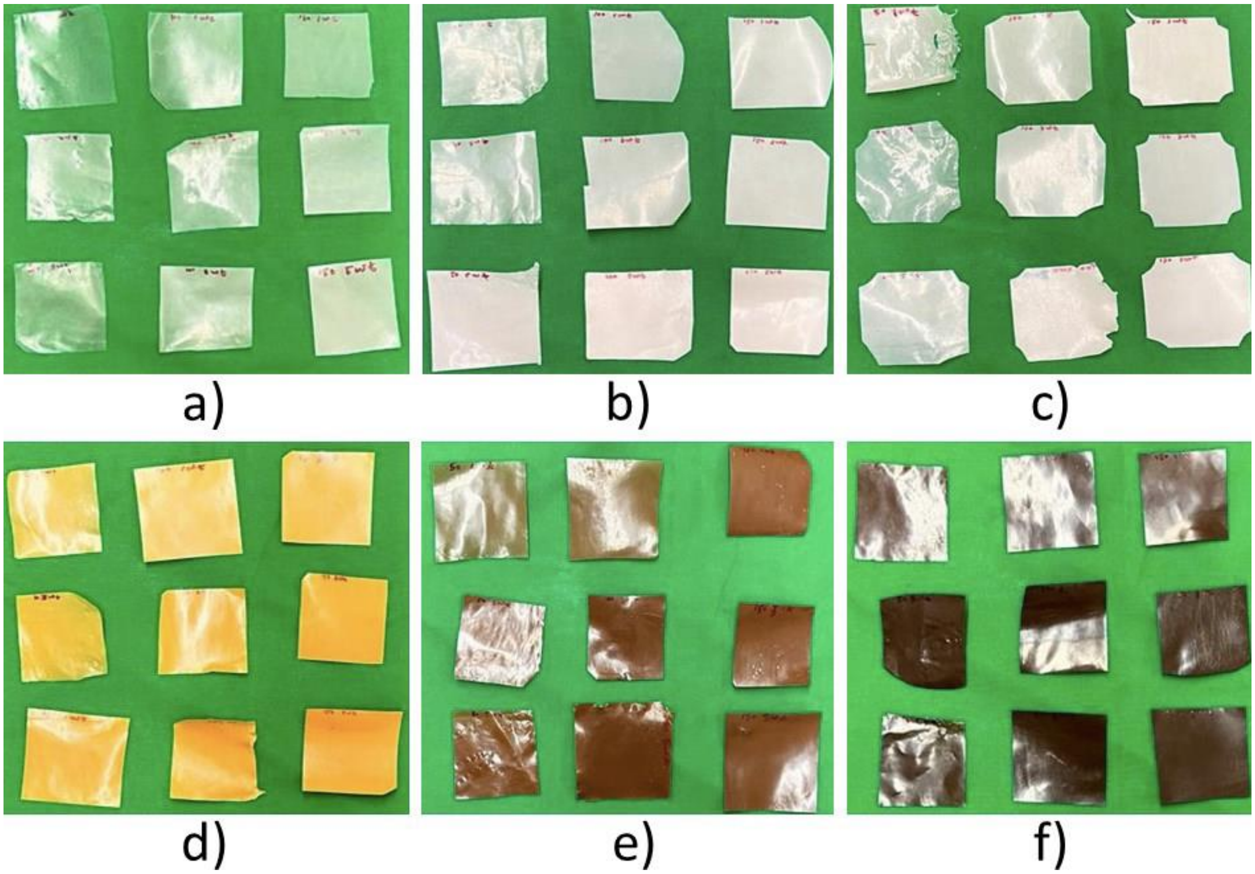

Figure S3: Pigmented LLDPE sheets with 50, 100, 150 μm thicknesses (vertical lines in the pictures) and 1, 3, 5 wt% loading concentrations (horizontal lines) for six pigments: a) ZnS, b) TiO_2, c) ZnO, d) FeO yellow, e) light brown, f) dark brown.

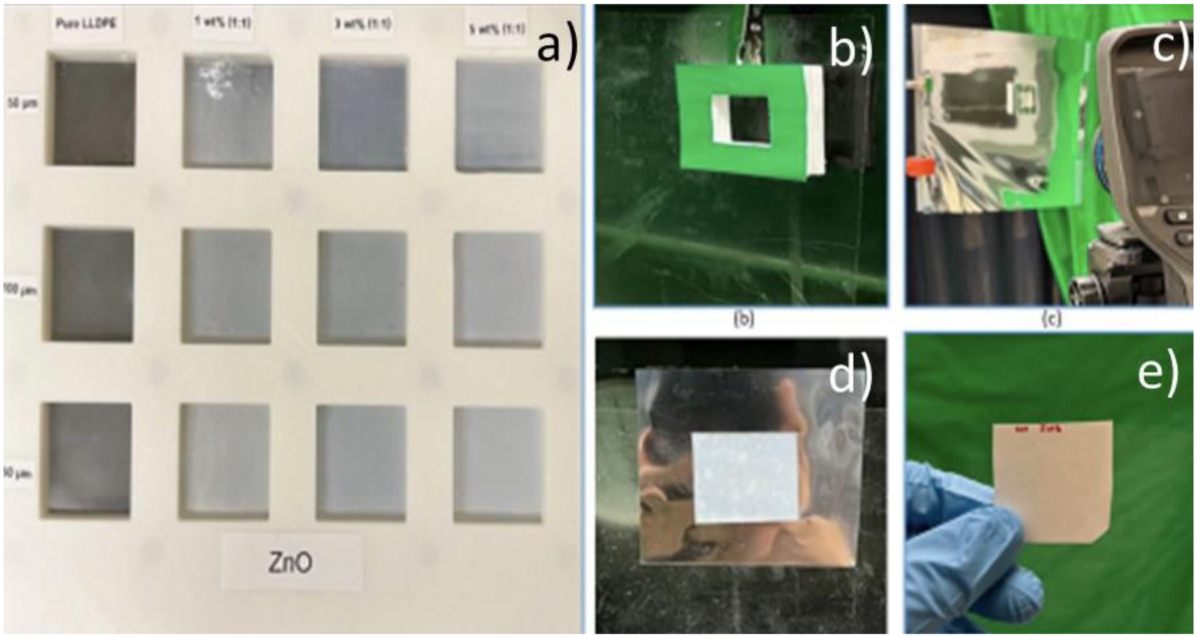

Figure S4: Setup for IR analysis. a) Fixture designed to hold the pigmented sheets, b) IR transparency setup with IR direct source, c) IR transparency setup with IR indirect source, d) Emissivity setup, e) sheet of ZnO-pigmented LLDPE.

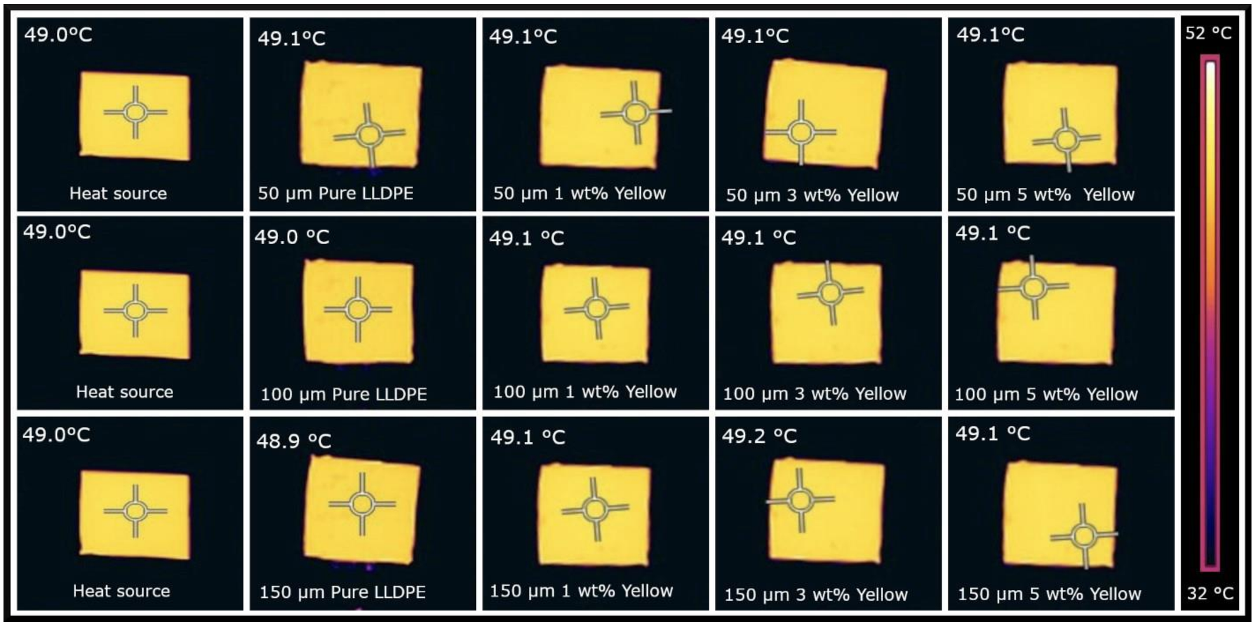

Figure S5: FeO yellow IR emissivity test for different sheet thicknesses (50,100,150um) and different loading concentrations (1, 3, and 5 wt%). Images for the heat source and pure LLDPE sheets at different thicknesses are provided as a comparison.

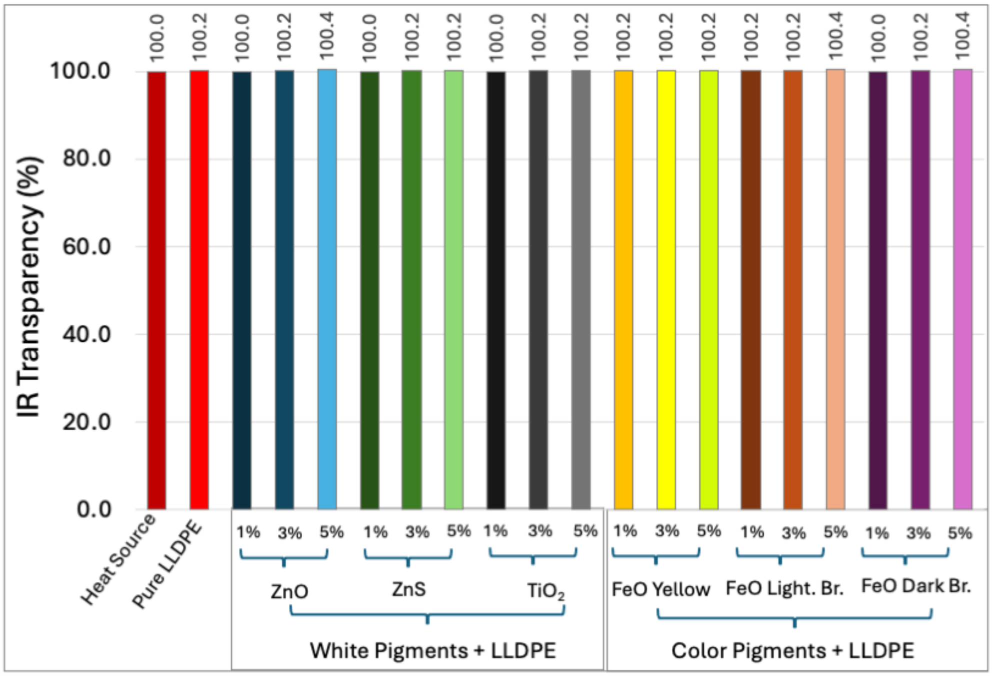
100.0
100.2
100.0
100.2
100.4
100.0
100.2
100.2
100.0
100.2
100.2
100.2
100.2
100.2
100.2
100.2
100.4
100.0
100.2
100.4
IR Transparency (%)
100.0
80.0
60.0
40.0
20.0
0.0
Heat Source
Pure LLDPE
1% 3% 5%
ZnO
1% 3% 5%
ZnS
1% 3% 5%
TiO2
White Pigments + LLDPE
1% 3% 5%
FeO Yellow
1% 3% 5%
FeO Light. Br.
1% 3% 5%
FeO Dark Br.
Color Pigments + LLDPE

(a)

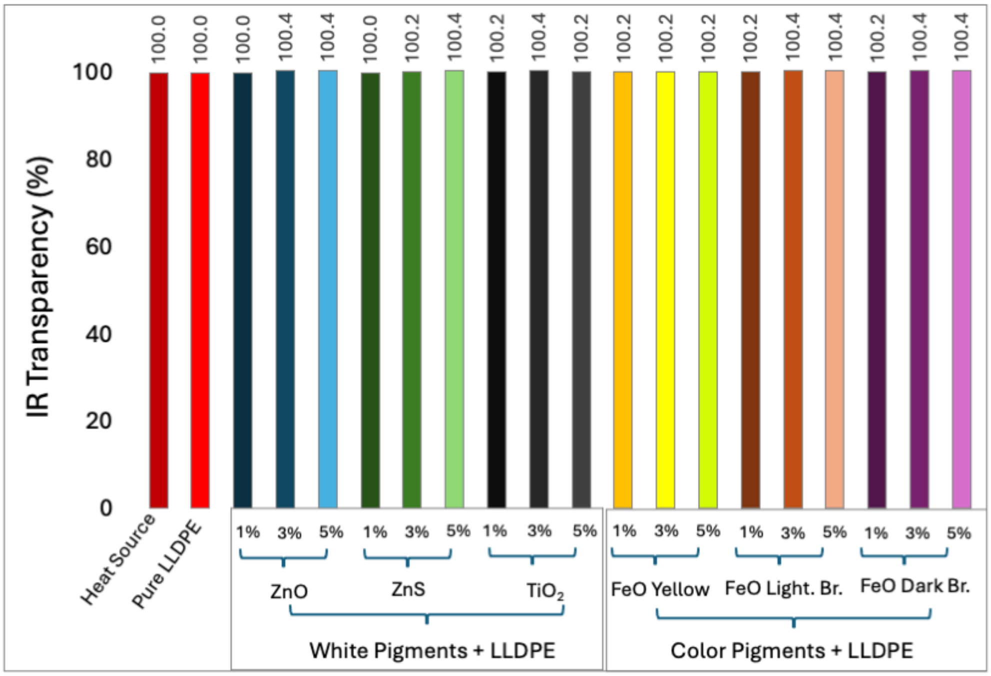
100.0
100.0
100.0
100.4
100.4
100.0
100.2
100.4
100.2
100.4
100.2
100.2
100.2
100.2
100.2
100.4
100.4
100.2
100.4
100.4
IR Transparency (%)
100
80
60
40
20
0
Heat Source
Pure LLDPE
1% 3% 5%
ZnO
1% 3% 5%
ZnS
1% 3% 5%
TiO2
White Pigments + LLDPE
1% 3% 5%
FeO Yellow
1% 3% 5%
FeO Light. Br.
1% 3% 5%
FeO Dark Br.
Color Pigments + LLDPE

(b)

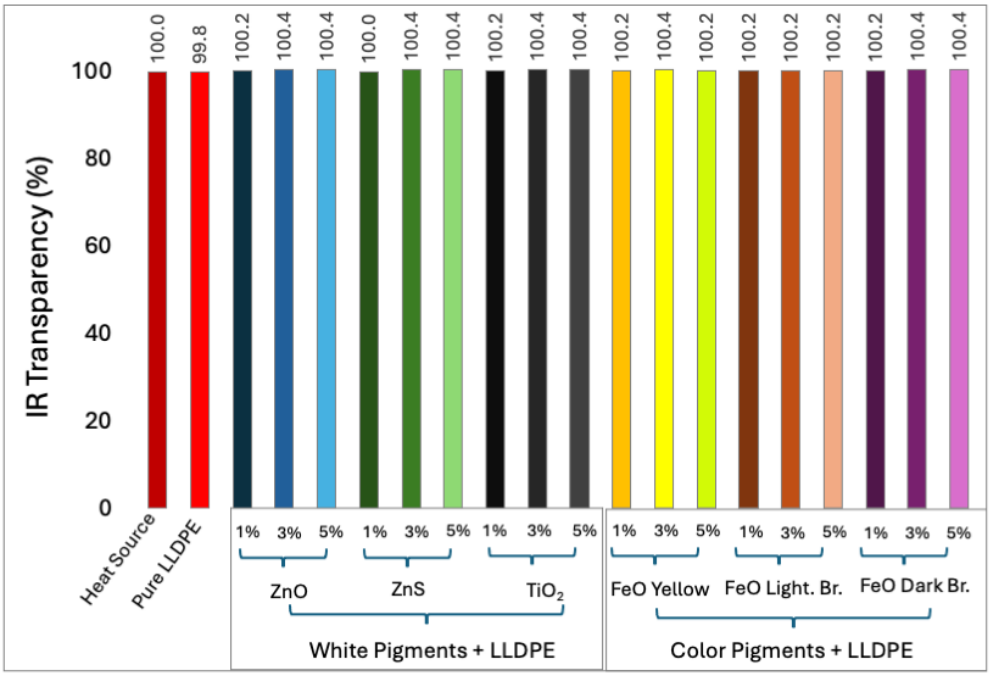

(c)

Figure S6: Results of the IR emissivity test: a) White pigments (ZnO, ZnS, TiO_2) at 50 µm, b) white pigments at 100 µm, c) white pigments at 150 µm, d) colored pigments (FeO yellow, light brown, dark brown) at 50 µm, e) colored pigments at 100 µm, and f) colored pigments at 150 µm
